# The Abdominal Ultrasonography Results of Cappadocia Cohort Study of Turkey Reveals High Prevalence of Fatty Liver

**DOI:** 10.5152/tjg.2023.23067

**Published:** 2023-06-01

**Authors:** Orhan Sezgin, Hale Akpınar, Birol Özer, Murat Törüner, Kadir Bal, Serhat Bor

**Affiliations:** 1 Department of Gastroenterology, Mersin University Medical Faculty, Mersin, Turkey; 2 Department of Gastroenterology, Dokuz Eylül University, İzmir, Turkey; 3 Department of Gastroenterology, Başkent University, Adana, Turkey; 4 Department of Gastroenterology, Ankara University Faculty of Medicine, Ankara, Turkey; 5 Department of Gastroenterology, İstanbul University-Cerrahpaşa, İstanbul, Turkey; 6 Department of Gastroenterology, Ege University Faculty of Medicine, İzmir, Turkey

**Keywords:** Cholelithiasis, community-based screening, fatty liver, gastroenterology, hepatic steatosis, mass screening, NAFLD, transabdominal ultrasonography

## Abstract

**Background:**

There is limited data about the prevalence of frequent gastrointestinal diseases in developing parts of the world based on community-based screening studies. Therefore, we aimed to present the detailed transabdominal ultrasonography results of the previously completed Turkey Cappadocia cohort study, which included a population-based evaluation of gastrointestinal symptoms and diseases in adults.

**Methods:**

This cross-sectional study was conducted in Cappadocia cohort. The transabdominal ultrasonography, anthropometric measurements, and disease questionnaires were applied to cohort persons.

**Results:**

Transabdominal ultrasonography was performed in 2797 subjects (62.3% were female and the mean age was 51 ± 15 years). Among them, 36% were overweight, 42% were obese, and 14% had diabetes mellitus. The most common pathological finding in transabdominal ultrasonography was hepatic steatosis (60.1%). The severity of hepatic steatosis was mild in 53.3%, moderate in 38.8%, and severe in 7.9%. Age, body mass index, liver size, portal vein, splenic vein diameter, hypertension, diabetes mellitus, and hyperlipidemia were significantly higher while physical activities were significantly lower in hepatic steatosis group. Ultrasonographic grade of hepatic steatosis was positively correlated with liver size, portal vein and splenic vein diameter, frequency of diabetes mellitus, hypertension, and coronary artery disease. Hepatic steatosis was observed in none of the underweight, 11.4% of the normal weights, 53.3% of the overweight, and 86.7% of the obese subjects. The percentage of hepatic steatosis cases with normal weight (lean nonalcoholic fatty liver disease) was 3.5%. The rate of lean nonalcoholic fatty liver disease in the entire cohort was 2.1%. Regression analysis revealed male gender (hazard ratio [HR]: 3.2), hypertension (HR: 1.5), and body mass index (body mass index: 25-30 HR: 9.3, body mass index >30 HR: 75.2) as independent risk factors for hepatic steatosis. The second most common ultrasonographic finding was gallbladder stone (7.6%). In the regression analysis, female gender (HR: 1.4), body mass index (body mass index: 25-30 HR: 2.1, body mass index >30 HR: 2.9), aging (30-39 age range HR: 1.5, >70 years HR: 5.8), and hypertension (HR: 1.4) were the most important risk factors for gallbladder stone.

**Conclusions:**

Cappadocia cohort study in Turkey revealed a high prevalence of hepatic steatosis (60.1%) while the prevalence of gallbladder stones was 7.6% among the participants. The results of the Cappadocia cohort located in central Anatolia, where overweight and lack of physical activity are characteristic, showed that Turkey is one of the leading countries in the world for nonalcoholic fatty liver disease.

**Main Points**

- We aimed to search gastrointestinal system health and the frequency of diseases by scanning the Cappadocia cohort that sampled Turkey with transabdominal ultrasonography.
- In this study, we determined that 60% of the population had liver steatosis.
- Regression analysis revealed male gender, hypertension (HT), and body mass index (BMI) as independent risk factors for hepatic steatosis (HS). Among the cases with HS, 3.5% had normal weight (lean NAFLD). The rate of lean NAFLD in the entire cohort was 2.1%.
- The second most common ultrasonographic finding was gallbladder stone (GBS) which was observed in 7.6% of the participants. In the regression analysis, female gender, BMI, aging, and HT were the most important risk factors for GBS.

## Introduction

The Cappadocia cohort is a cohort of approximately 10 000 people living in Gülşehir and Avanos districts of Nevşehir Province in the Central Anatolian region of Turkey. Gastrointestinal (GI) symptoms and burden of GI disease in the adults of this cohort were previously published in a study conducted by us, and the frequencies of upper gastrointestinal system (GIS) disease and lower GIS disease were reported as 33% and 12%, respectively.^1^ This study showed that Turkey is one of the leading countries in Europe in terms of obesity.^2,3^ Considering the high prevalence of obesity and diabetes mellitus (DM) in Turkey, it is important to evaluate and monitor the liver and pancreato-biliary system for possible disorders. It is very valuable to establish community-based screening programs in order to reach conclusions and develop strategies for public health. Therefore, we evaluated the results of the volunteers who participated in the previous Cappadocia cohort study who underwent transabdominal ultrasonography (TAU). Transabdominal ultrasonography is a simple, economical, noninvasive, and reproducible examination method with proven efficacy and reliability, preferred as the first imaging method in the evaluation of all intra-abdominal solid-parenchymatous and canalicular organs. For this purpose, it is frequently used in community surveys.^4,5^ In this study, we aimed to evaluate the main disorders of the liver, biliary system, pancreas, spleen, and vascular structures in a large sample group representing Turkey.

## Materials and Methods

The study is a prospective cross-sectional cohort study and was conducted in the “Cappadocia Cohort” consisting of Avanos and Gülşehir districts of Nevşehir, a geographical region that has been shown to represent Turkey exactly in terms of population distribution. These districts were previously selected by the Turkish Internal Medicine Specialization Association due to their low migration rates and geographical proximity to the capital Ankara, and some studies were carried out.^6^ Another factor in the selection of these 2 districts was that they did not have a major economic or social dependency. The economy of Gülşehir district is based on agriculture, and the economy of Avanos district is mostly dependent on tourism. Written permission for the study was obtained from Dokuz Eylül University Non-Interventional Research Ethics Committee (Number 363, Date: April 12, 2018) and Nevşehir Provincial Health Directorate.

Volunteer adults aged 18 and over living in Avanos and Gülşehir between October 2017 and July 2018 were included in the study. In both districts, the district health directorate, district governorships, municipalities, and family health centers were informed and the study was announced in the districts. In this context, information and invitation brochures and posters on the subject were prepared and their distribution and announcements were made at the central points of the districts. Those who accepted to participate in the study were invited to the study offices to perform TAU with their physical measurements. Measurements were made at the Turkish Internal Medicine Specialization Association study office for Gülşehir district and Avanos District Integrated Hospital for Avanos district. After obtaining written consent, physical measurements of the volunteers were done by the interviewers at the study office. Demographic data including age and gender, alcohol use and its amount (≥14 units/week in women, ≥21 units/week in men), the presence of comorbid diseases, duration of physical activity at work and leisure time,^7^ and the number of births in women were questioned. Height (m), body weight (kg), body mass index (BMI), and waist circumference (cm) were measured for anthropometric assessment. Hypertension (HT) was defined as a systolic blood pressure above 130 mm Hg, and/or diastolic blood pressure above 85 mm Hg. Body mass index was evaluated in 4 groups as <18 kg/m^2^ (thin), 18-24.9 kg/m^2^ (normal), 25-29.9 kg/m^2^ (overweight), and ≥30 kg/m^2^ (obese). Transabdominal ultrasonography was performed by a single experienced radiologist.

### Transabdominal Ultrasonographic Examination

The transabdominal ultrasonographic examinations were performed using a high-resolution ultrasonography equipment (Toshiba Aplio 80, Japan) with 1-6 MHz convex transducer. To avoid the interobserver variability, the ultrasonography in this work was performed by a single radiologist. The radiologist meetings with the experienced members of the study group in ultrasonography (OS, BÖ) agreed on the definition and images. The radiologist was blind to the patients’ questionnaires or medical histories. Transabdominal ultrasonography was performed in the morning, following at least 8 hours of fasting, with the subject in supine position and with the abdomen fully open. All intra-abdominal vascular formations, parenchymatous organs (liver, pancreas, spleen, kidneys), hollow organs (gallbladder, bladder), and GI tract were evaluated by scanning from transverse, sagittal, and oblique sections.^8^

With TAU, subcostal and intercostal sections, size of the liver (craniocaudal length of the liver by measuring the upper–lower border of the liver in the right midclavicular and even longitudinal section), marginal arrangement (regular or irregular), parenchyma structure (homogeneous or heterogeneous), and echogenicity (normal, increased, decreased), left corner angle (sharp or blunt), and the presence of a mass in the parenchyma were evaluated. The normal craniocaudal length of the liver was considered to be below 15 cm. If a mass was detected in the liver, its characteristics and size were determined. Portal vein (PV), splenic vein, hepatic veins, and inferior vena cava diameters, luminal openings, wall features, and the presence of any obstruction to flow were determined.

Liver parenchymal echogenicity was evaluated by comparing to the echogenicity of the kidney and spleen. Liver parenchymal echogenicity was considered normal when liver and kidney echogenicity were equal. If the kidney parenchyma was pathologic, liver echogenicity was compared to the echogenicity of the spleen. If liver parenchymal echogenicity was higher than the echogenicity of the kidney, hepatic steatosis (HS) was diagnosed.^9-11^ Hepatic steatosis was graded according to echo patterns as follows: grade 0, normal liver echotexture; grade 1, slight and diffuse increase in parenchymal echotexture with normal visualization of the diaphragm and intrahepatic vessel borders; grade 2, moderate and diffuse increase in parenchymal echotexture with slightly impaired visualization of the diaphragm and intrahepatic vessel borders; and grade 3, significant increase in parenchyma echotexture with poor visualization of the diaphragm and intrahepatic vessel borders.^9-11^

The structure of the gallbladder, thickness of its wall, and the presence of a formation in the lumen of the gallbladder were examined in longitudinal, oblique, and transverse sections.^12,13^ The thickness of the gallbladder wall was measured from the position where the ultrasound beams were perpendicular to the gallbladder wall. Gallbladder wall thickness greater than 3 mm was considered as increased gallbladder wall thickness. The formations in the gallbladder with hyperechoic and posterior acoustic shadow were considered as stones. Nonmoving iso- or hyperechoic formations associated with the gallbladder wall and without acoustic shadow were considered as gallbladder polyps. The presence of homogeneous or irregular thickening in the gallbladder wall was also evaluated. Gallbladder size was evaluated by measuring the longitudinal and transverse diameters. Hydropic gallbladder was considered in the presence of a longitudinal diameter exceeding 10 cm and/or transverse diameter exceeding 5 cm. The presence of dilation in the intrahepatic bile ducts was also recorded.

The pancreas was examined with transverse and sagittal movements of the transducer placed in the epigastric region. The head, body, and tail of the pancreas were evaluated. The parenchymal structure, echogenicity, homogeneity, thickness, and presence of a mass and ductal structure of the pancreas were evaluated.^8^ The normal thickness of the pancreas is approximately 2-2.5 cm in the head and 2 cm in the body, and the normal diameter of the pancreatic duct is up to 3 mm in the head region.

In TAU, spleen size (craniocaudal length), parenchyma structure, and presence of any formation were evaluated.^8^ A craniocaudal length of spleen up to 12 cm and the maximum thickness of 5 cm was considered to be normal. The parenchyma structure should be homogeneous and isoechoic with normal liver parenchyma.

The location and size of the kidneys, parenchyma and collecting system structures,^14^ bladder and abdominal aorta diameter, wall structure and branches were evaluated.^15^

Those who were found to have pathological findings that required further investigation in TAU were recommended to apply to the hospital by giving a standard result and recommendation form.

### Statistical Analysis

The program Predictive Analytics SoftWare (PASW) Statistics Version 18.0 for Windows (SPSS Inc., Chicago, IL, USA) was used for statistical analysis. Descriptive statistics were used as numbers and percentages for categorical variables, and mean ± standard deviation, median and percentile 25-75 (Q1–Q3) for numerical variables. The conformity of the variables to the normal distribution was examined using visual (histogram and probability graphs) and analytical methods (Kolmogorov–Smirnov/Shapiro–Wilt tests). Chi-square analysis was used in the analysis of categorical variables of TAU findings and in pairwise and multiple comparisons, and Fisher’s exact test was used when the chi-square condition was not met. Student’s *t*-test was used in paired group comparisons and ANOVA was used in multiple group comparisons when the normal distribution condition was met with numerical variables; If the normal distribution condition was not met, Mann–Whitney *U* test was used for paired group comparisons and Kruskal–Wallis test was used for multiple group comparisons. Logistic regression analysis was performed by creating a model with gender, age, BMI, and comorbid disease parameters to determine the risk factors of TAU findings. Cases with a type 1 error level below 5% were considered statistically significant.

## Results

**Figure 1.**
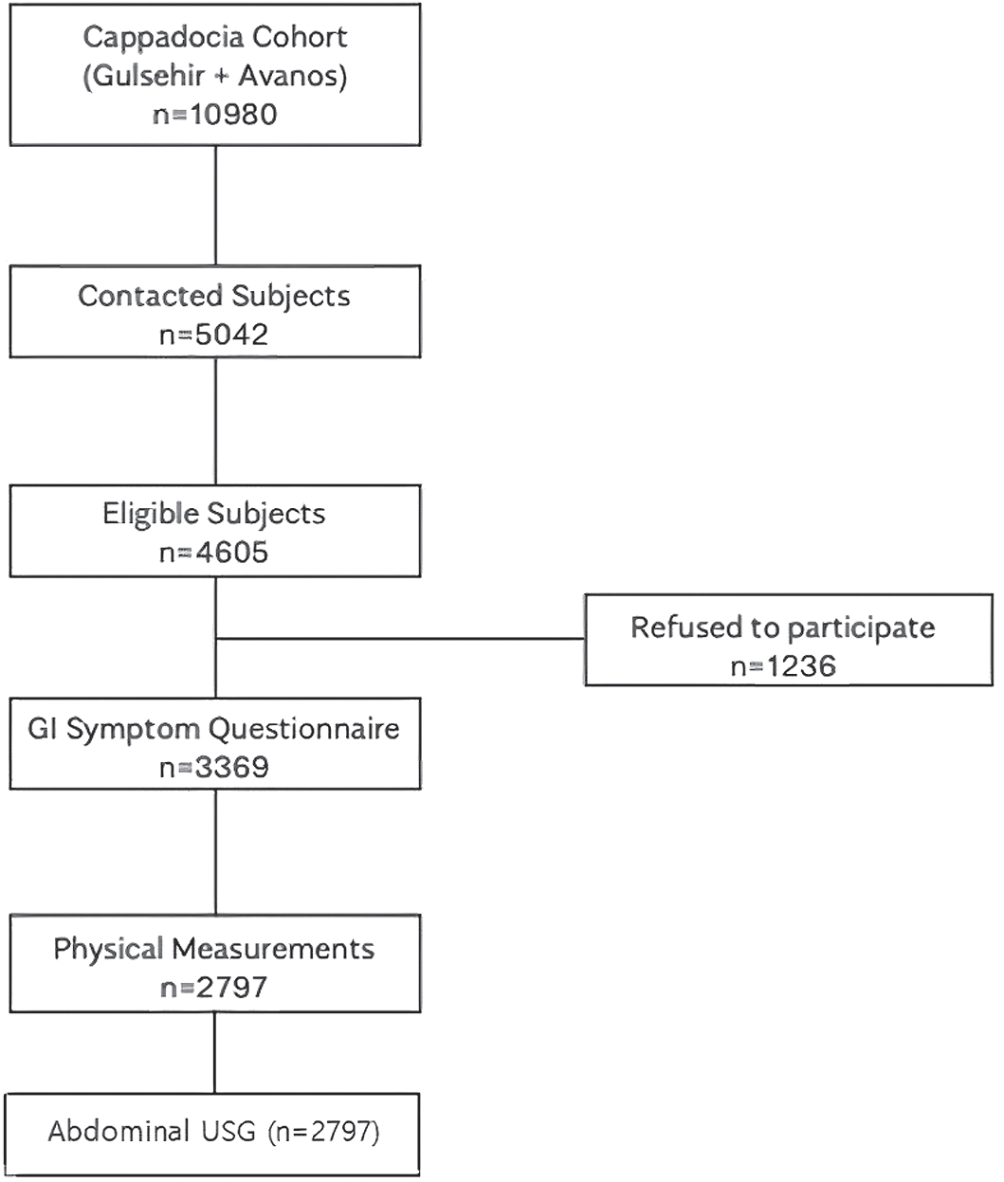
Transabdominal ultrasonography was performed in the Cappadocia cohort.

The total population of Avanos and Gülşehir districts was 25 502 in 2018. In the current study, out of 10 980 people in the Cappadocia cohort, 5042 were contacted, and a GI Symptom Questionnaire was applied to 3369 among the 4605 subjects who met the inclusion criteria. The remaining 1236 people refused to participate in the study.^1^ And 2797 out of 3369 people who filled out the survey agreed to have anthropometric measurements (height and weight) and TAU (Figure 1).

The mean age of the 2797 volunteers who underwent TAU was 52 ± 14.5 years, 62.4% were female and 37.6% were male. Mean BMI was 29.1 ± 5.4 kg/m^2^, and 78.1% were overweight (36.4%) or obese (41.7%). The mean waist circumference was 96.8 ± 13.8 cm. Comorbid diseases of the volunteers were HT (38.9%), DM (14.0%), hyperlipidemia (HL)/hypercholesterolemia (8.3%), coronary artery disease (CAD; 7.8%), chronic kidney disease (0.4%), and cancer (0.3%). Six percent of the volunteers had a history of alcohol use. The physical activity of the volunteers was mild to moderate in 95.8%. The mean number of deliveries in women was 2.8 ± 1.4.

### Ultrasonographic Evaluation of the Liver

The mean size of the liver of the volunteers was 150 ± 9.2 mm and it was normal in 64.5%, increased in 35.4%, and decreased in 0.1% of the cases. Liver echogenicity was increased in 60.1% of the cases which was compatible with HS. Of those with HS, 53.3% had mild steatosis, 38.8% had moderate, and 7.9% had severe steatosis. Liver parenchyma structure was homogeneous in 99.2% of the volunteers.

A mass in the liver was detected in 2.5% (n = 69) of the volunteers. Eighty-four percent (n = 58) of these masses were compatible with typical hemangioma. In 5 cases (7.2% of masses, 0.1% in the whole cohort), there were masses which met the malignancy criteria. In 6 cases, the distinction could not be made. Liver cyst was detected in 3% and cirrhosis findings in 0.2%. The mean PV diameter was 11 ± 1.1 mm, the splenic vein diameter was 7 ± 0.8 mm. Hepatic veins and inferior vena cava were evaluated as normal in all of the volunteers. The above-mentioned pathologies of the patients were not previously diagnosed.

### Hepatic Steatosis

Hepatic steatosis was detected in 65.1% of the men and 57% of the women in the Cappadocia cohort (*P* < .001). Volunteers with HS were significantly older than those without HS (mean age 55 ± 12 years vs. 45 ± 16 years, *P* < .001) and had a higher BMI (32.2 ± 4.9 vs. 25.8 ± 4.0, *P* < .001) and liver size (159.7 ± 14.7 mm vs. 137.3 ± 11.6 mm, *P* < .001). The frequency of HS according to age groups in this cohort was 19.1% in the volunteers <30 years, 35.5% in the volunteers between 30 and 39 years, 58.3% in those between 40 and 49 years, 73.0% in volunteers between 50 and 59 years, 76.7% between 60 and 69 years, 68.9% between 70 and 79 years, and 53.4% in the volunteers >80 years (*P* < .001). On the other hand, 2.6% of those with HS were <30 years, 9.4% were between 30 and 39 years, 18.3% between 40 and 49 years, 34.1% between 50 and 59 years, 25.1% between 60 and 69 years, 9.1% were between 70 and 79 years, and 1.4% were older than 80 years.

The frequency of DM (19.4% vs. 5.2%), HT (46.8% vs. 26.0%), and HL (10.5% vs. 4.7%) was significantly higher in patients with HS compared to those without HS (*P* < .001). There was no significant difference between the groups in terms of the presence of CAD (8.6% vs. 6.4%, *P* = .109). The mean number of births in the women with HS was significantly higher than in the women without HS (3 vs. 2, *P* < .001). Physical activity at work (*P*: .247) and at leisure time (*P* = .117) was lower in those with and without HS.

**Figure 2.**
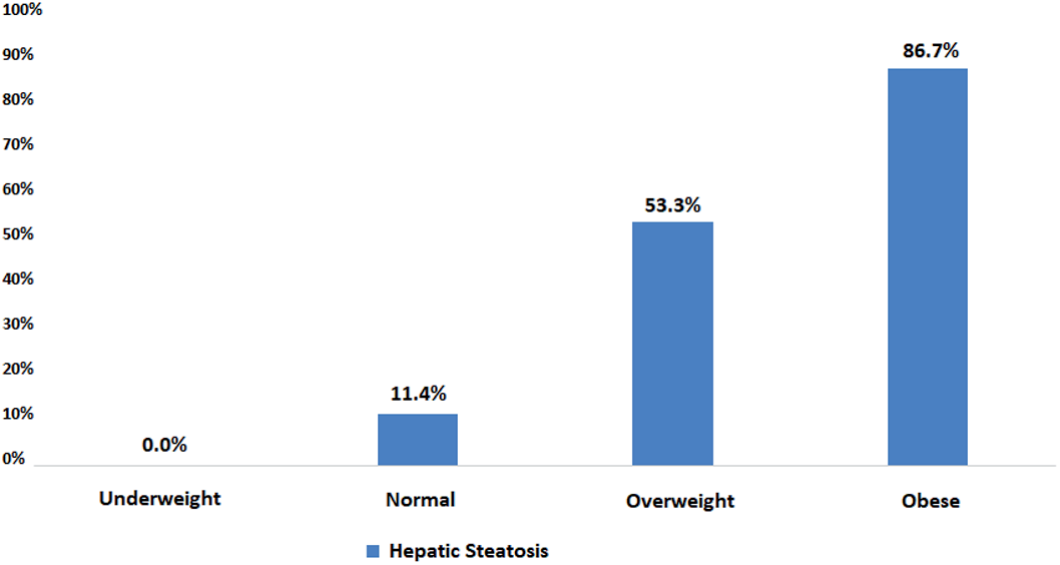
Frequency of hepatic steatosis according to BMI. BMI, body mass index.

According to BMI values, HS was observed in 0% of the thin (n = 0), 11.4% of the normal (n = 517), 53.3% of the overweight (n = 971), and 86.7% of the obese individuals (n = 1247; Figure 2). The frequency of HS increased significantly as BMI increased (*P* < .001). The mean BMI of the volunteers with HS was <25 in 3.6%, between 25 and 30 in 31.2%, and >30 in 65.2% (*P* < .001).

**Figure 3.**
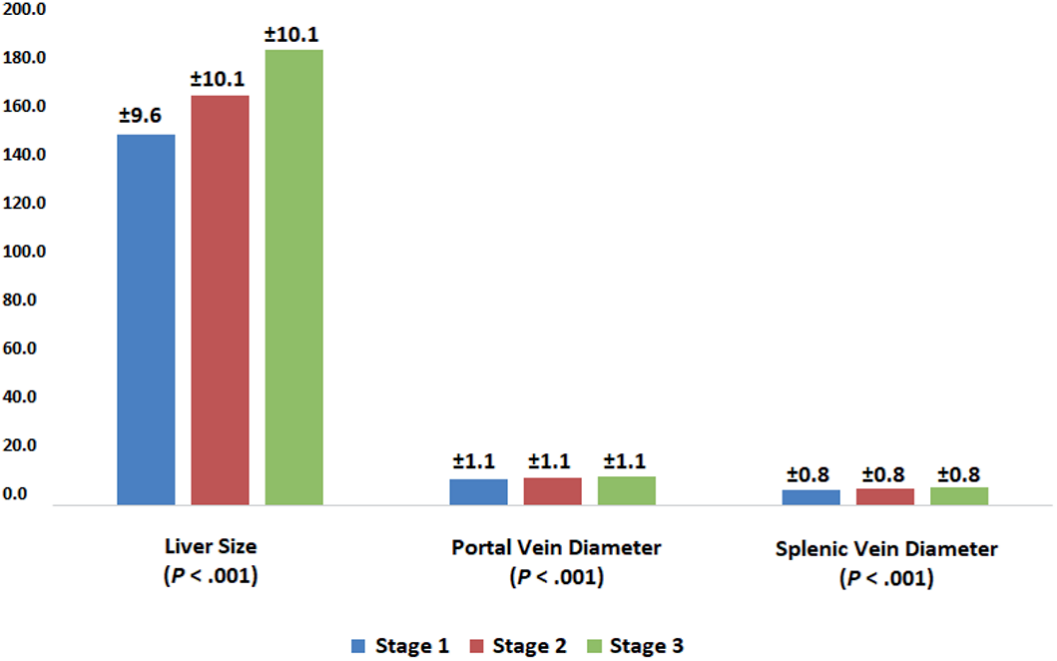
Measurements according to the hepatic steatosis stages.

The liver size was 150.8 ± 9.6 mm in subjects with grade 1 HS, 166.7 ± 10.0 mm in grade 2 HS, and 185.2 ± 10.0 mm in grade 3 HS. As the HS grade increased, there was a significant increase in liver size (*P* < .001; Figure 3). When PV diameter was evaluated, according to the grade of HS, it was 10.7 ± 1.1 mm in grade 1 HS, 11.0 ± 1.1 mm in grade 2, and 11.7 ± 1.1 mm in grade 3 (*P* < .001 for all). Splenic vein diameters were 6.4 ± 0.8 mm, 6.7 ± 0.8 mm, and 7.2 ± 0.8 mm in grades 1, 2, and 3 HS, respectively (*P* < .001 for all; Figure 3). Portal vein and splenic vein diameters increased in correlation with HS grade.

**Figure 4.**
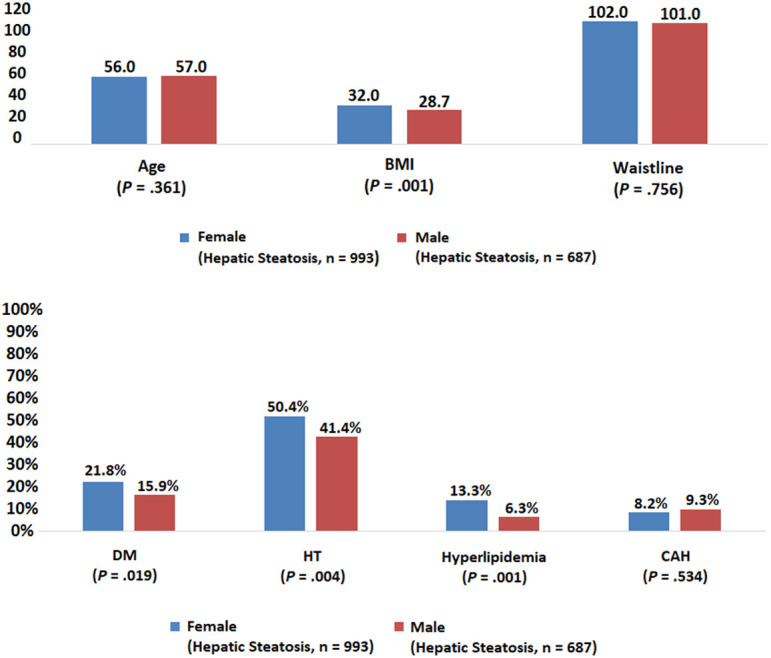
Evaluation by gender in patients with hepatic steatosis.

When the volunteers with HS were compared with respect to gender, females had a significantly higher BMI (32.0 vs. 28.7, *P* = .001) and a higher frequency of DM (21.8% vs. 15.9%, *P* = .019), HT (50.4% vs. 41.1%, *P* = .004), and HL (13.3% vs. 6.3%, *P* = .001) compared to males (Figure 4). There was no significant difference between the waist circumference, frequency of CAD, and physical activity status of the female and male volunteers.

**Figure 5.**
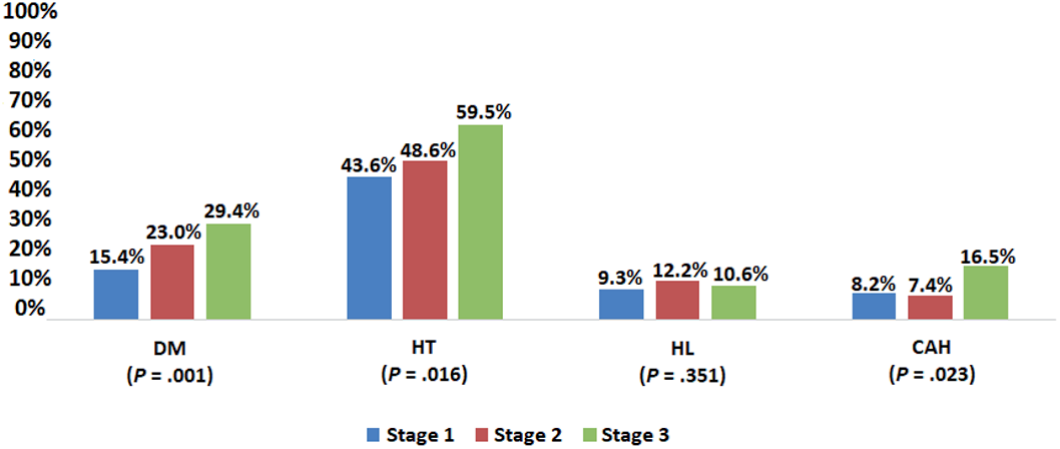
Frequency of comorbidities according to hepatic steatosis stage.

When the presence of comorbid diseases was evaluated according to the grade of HS, DM was seen in 15.4% of the volunteers with grade 1 HS, 23% in grade 2, and 29.4% in grade 3 (*P* = .001). The frequency of HT was 43.6%, 48.6%, and 59.5%, in the same group, respectively (*P* = .016). Hyperlipidemia was seen in 9.3% of the volunteers with grade 1 HS, in 12.2% of the volunteers with grade 2 HS, and in 10.6% of the volunteers with grade 3 HS (*P*: .351). Finally, the frequency of CAD was 8.2%, 7.4%, and 16.5% in volunteers with grades 1, 2, and 3, respectively (*P* = .023; Figure 5). The number of deliveries in women increased parallel to the increase in the grade of HS (2.9 ± 1.5, 3.2 ± 1.4, 3.2 ± 1.3, respectively, *P* = .007). No significant relationship was found between HS and physical activity. While 4.2% of the cohort was very active, 95.8% were mild-moderately active. Even physical activity at work was very little in 28.3% and moderate in 61.8%.

Body mass index was <25 in 3.5% of those with HS. This group was named as lean HS. While the frequency of lean HS was 5.8% in male volunteers with HS, it was 1.9% in females (*P* < .001). The mean age of volunteers with lean HS (50.34 ± 13.91, range: 39-62) was significantly lower than the non-lean (BMI > 25) volunteers (54.77 ± 12.19, range: 47-63; *P* = .019). When the grades of HS were evaluated in lean and non-lean HS volunteers, steatosis was grade 1 in 74.6% of the lean and 52.8% of the non-lean volunteers, grade 2 in 20.3% of the lean and 39.3% of the non-lean volunteers, and grade 3 in 5.1% of the lean and 7.9% of the non-lean HS volunteers (*P* = .004). Liver size was 154.15 ± 11.35 mm versus 159.82 ± 14.75 mm (*P* = .003), PV diameter was 10.64 ± 1.32 mm versus 10.91 ± 1.14 mm (*P* = .091), and splenic vein diameter was 6.39 ± 1.26 mm versus 6.60 ± 0.79 mm (*P* = .012).

When the risk factors for HS were evaluated with regression analysis, male gender (HR: 3.2), presence of HT (HR: 1.5), and high BMI (BMI: 25-30 HR: 9.3, BMI > 30 HR: 75.2) were found to be independent risk factors for HS (Table 1).

### Ultrasonographic Evaluation of the Gallbladder

Gallbladder was normal in 81.4% of the cohort. The median gallbladder wall thickness of the Cappadocia cohort was 3 mm, and no pathological wall structure was detected. Of the volunteers, 7.6% had gallbladder stones (GBSs), 2.1% had gallbladder polyps, 1 person had an image compatible with Caroli’s disease, and 1 had a stone in the intrahepatic bile duct.

### Gallbladder Stone

**Figure 6.**
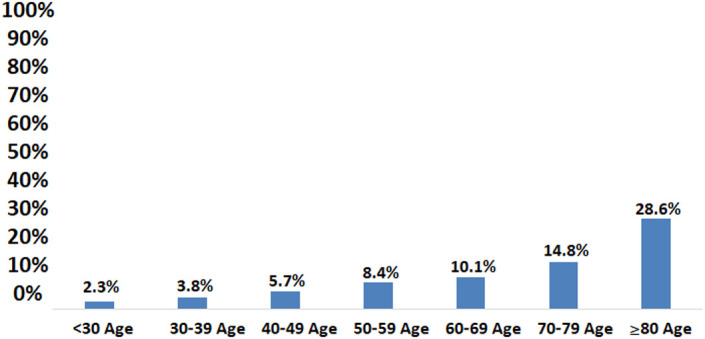
The relationship between age and gallstone.

Gallstones were detected in 7.6% of the volunteers. Gallstones were detected in 6.2% of the men and 8.5% of the women participated in the Cappadocia cohort (*P* = .033). The median size of the gallstone was 12 mm. Of those with gallstones, 32.5% were men and 67.5% were women (*P* = .033). Age (57 ± 14, median 58 vs. 49 ± 14, median 51) and BMI (31.60 ± 5.49, median 31.21 vs. 29.25 ± 5.54, median 28.74) were significantly higher in those with gallstones than those without (*P* < .001). Of those with GBSs, 2.6% were <30 years, 8.2% were 30-39 years, 14.4% were 40-49 years, 31.4% were 50-59 years, 24.2% were 60-69 years, 13.9% were between 70 and 79 years, and 5.2% were older than 80 years. The incidence of gallstones was 2.2% under the age of 30, 3.7% in the 30-39 age range, 5.6% in the 40-49 age range, 8.4% in the 50-59 age range, 10% in the 60-69 age range, and it was 14.7% between 70 and 79 years, and 28.5% in those older than 80 years (*P* < .001; Figure 6).

**Figure 7.**
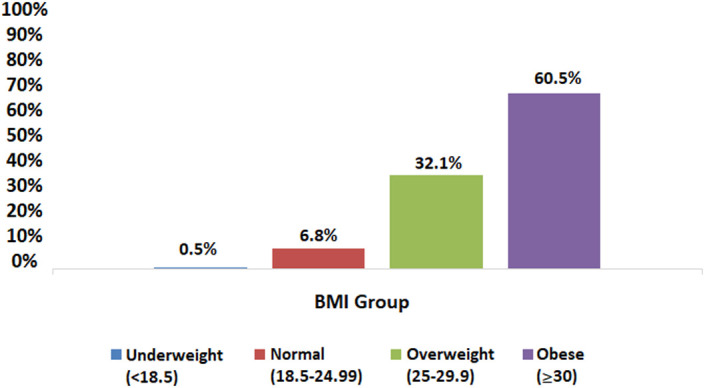
Distribution of gallstones by BMI. BMI, body mass index.

When the relationship between BMI and gallstone frequency was evaluated, the BMI of those without stones was 29.2 ± 5.5 kg/m^2^, while it was 31.6 ± 5.4 kg/m^2^ in those with stones (*P* < .001). Of those with GBSs, 0.5% were underweight, 6.8% were normal, 32.1% were overweight, and 60.5% were obese (*P* < .001; Figure 7). As the BMI increased, the incidence of gallstones increased. The frequency of gallstones was 2.6% in those with a BMI < 25, 6.8% in those with a BMI of 25-30, and 10.6% in those with a BMI > 30 (*P* < .001).

Patients with GBSs had a higher frequency of DM (23.3% vs. 13.2%, *P* = .001), HT (48.1% vs. 29.3%, *P* < .001), HL (13.8% vs. 7.0%, *P* = .007), and CAD (12.9% vs. 7.0%, *P* = .02) compared to those without gallstones. There was no difference between the patients with and without GBSs in terms of physical activity at work and in the leisure time. When the 194 volunteers with GBSs were evaluated according to their gender; mean BMI was higher in women (31.5%) than men (29.3%) (*P* = .030). Waist circumference was higher in men than in women (107 cm vs. 101 cm, *P* = .041). Age was similar between the genders (male 61 ± 11 years, female 56 ± 15 years, *P* = .383). The frequencies of other comorbid diseases, such as DM (18.6% vs. 24.7, *P*: .450), HT (60.5% vs. 53.4, *P*: .461), HL (11.6% vs. 15.1%, *P* = .604), and CAD (14% vs. 12.3%, *P* = .801) were not significantly different in men and women, respectively. No significant difference was found between women with and without GBSs in terms of the number of births (*P* = .069).

Regression analysis revealed female gender (HR: 1.4), increased BMI (BMI: 25-30 HR: 2.1, BMI > 30 HR: 2.9), aging (30-39 age range HR: 1.5, >70 years HR: 5.8), and HT (HR: 1.4) as independent risk factors for the presence of gallstones (Table 2).

### Gallbladder polyps

Gallbladder polyps were detected in 2.1% of the individuals in the cohort. In the Cappadocia cohort, 2.5% of men and 1.9% of women had gallbladder polyps (*P* = .313). Of those with polyps in the gallbladder, 46.3% were men and 53.7% were women (*P* = .620). There was no significant difference between the subjects with and without gallbladder polyps with respect to age (47 ± 14 vs. 50 ± 15, *P* = .139), BMI (29.5 ± 6.5 vs. 29.4 ± 5.5 kg/m^2^, *P* = .362), waist circumference (88.9 ± 11.1 vs. 92.2 ± 13.8, *P* = .149), presence of concomitant comorbid diseases, such as DM (11.1% vs. 12.8, *P* = 1.000), HL (0.0% vs. 7.6, *P* = .258), CAD (3.7% vs. 7.6, *P* = .716), physical activity status (*P* = .628), and the number of births in women (*P* = .899). Hypertension was significantly more common in volunteers without gallbladder polyps (38.0%) compared to those with polyps (14.8%; *P* = .014).

There was no difference in any parameter according to gender in those with polyps in the gallbladder. There was no relationship between BMI and polyp in the gallbladder. Gallbladder polyps were observed in none of those with a BMI < 18, in 1.8% of those with a BMI < 25%, in 3.0% of those with a BMI between 25 and 30, and in 1.7% of those with a BMI > 30 (*P* = .163). There was no relationship between age and the presence of gallbladder polyps, which were observed in 3.2% of those <30 years, 2.1% of those between 30 and 39 years, 3.0% of those between 40 and 49 years, 1.8% of those between 50 and 59 years, 1.3% of those between 60 and 69, 1.6% of those between 70 and 79 years, and in 2.9% of those >80 years (*P* = .478).

### Cholecystectomy

Two hundred fifty (8.9%) individuals in the Cappadocia cohort had undergone cholecystectomy. Women had a higher rate of cholecystectomy history (11.8%) than men (4.3%; *P* < .001). Since the information and indications of cholecystectomy at the time of those who had cholecystectomy could not be evaluated, we did not make any further evaluation on this subject.

### Ultrasonographic Evaluation of the Pancreas

In the ultrasonographic evaluation, the pancreas was evaluated as normal in 99.9% of the volunteers (n = 2794). It was atrophic in only 2 subjects (0.1%). Sonographic evaluation revealed no mass and cyst was detected in 1 case. Parenchymal echogenicity was increased in 1 case and the others were evaluated as normal and homogeneous. The mean body thickness was 16 ± 5.1 mm.

### Ultrasonographic Evaluation of the Spleen

The spleen was ultrasonographically normal in 99.7% (n = 2788) of the volunteers. The mean craniocaudal length of the spleen was 100 ± 7.3 mm. Splenomegaly was found in 18 volunteers, cysts in 5, and masses in 2 volunteers. One person had a history of splenectomy. However, the possible etiology could not be determined.

### Ultrasonographic Evaluation of the Vascular Structures

The abdominal aorta was ultrasonographically normal in 99.5% of the volunteers (n = 2783). Aneurysm was found in 11 (0.4%) patients. Aortic thrombus was detected in 1 case. The median aorta diameter was 16 ± 9.3 mm.

## Discussion

The Cappadocia cohort is a cohort located in the center of Turkey and exemplifies Turkey in terms of population ratio. But this study exemplifies adult subjects over 18 years old. In the first study we conducted with a cohort, we found that 70% of the population had GIS complaints, 33% had upper GIS disease, 12% had lower GIS disease, and 10% had a combination of both.^1^ In addition, the mean BMI of the cohort was 29.7 ± 5.6 kg/m^2^. Approximately 35.2% were overweight and 45.2% were obese. This study has definitely shown that GIS diseases, overweight, and obesity have an important place among the health problems of our country. Especially in such a population, it is important to evaluate the hepato-bilio-pancreatic system and the entire abdomen with abdominal imaging. On the other hand, there are no comprehensive, population-based studies on ultrasonographic results of the digestive system in our country. In addition, it is extremely difficult to conduct studies that sample the whole country. For this reason, choosing a region that is cross-sectionally representative of the country is a method that enables the findings to be generalized.

In the evaluation with TAU in the Cappadocia cohort, the most common finding was HS (60.1%). Hepatic steatosis can be due to many reasons; some of these are alcohol intake, DM, obesity, drug use, metabolic syndrome, and aging. Since alcohol consumption was 6% in the Cappadocia cohort, we can say that the HS we detected is due to nonalcoholic fatty liver disease (NAFLD). As a general definition, in nonalcoholic fatty liver, in the presence of liver steatosis on ultrasonography, daily intake of alcohol should be less than 30 g/day for men and less than 20 g/day for women and the absence of any other liver disease. Transabdominal ultrasonography is a widely accepted basic diagnostic method for determining HS and grading the amount of fatty infiltration. It has been proven in many studies that HS and its degree detected by ultrasonography are associated with metabolic syndrome.^16-18^

The estimated worldwide prevalence of NAFLD ranges from 20% to 46%.^19^ The rate of NAFLD detected in the Cappadocia cohort was extremely high at 60%. In the United States, NAFLD has been estimated to affect approximately 30% of the population. However, the prevalence is especially higher among obese (70%) and diabetic (90%) individuals.^20^ The worldwide spread of sedentary lifestyle and Western-type diet plays a role in the increase of NAFLD in both children and adults in many countries.^21-23^ In a meta-analysis involving 8.5 million people from 22 countries, it was shown that more than 80% of nonalcoholic steatohepatitis (NASH) patients were overweight or obese, 72% had dyslipidemia, and 44% had type 2 DM.^24^ Nonalcoholic fatty liver disease/NASH is a disease at the crossroads of gastroenterology, cardiovascular diseases, endocrinology/metabolism diseases, and oncology.

In studies conducted in Turkey about this topic, in 2006, the prevalence of NAFLD by abdominal USG was found to be 19.8% in overall, 36.4% for people >50 years of age, and 7.9% for people between 18 and 29 years of age with 404 healthy adults in Elazığ, which is a rural area in Turkey.^25^ More recently, in 2016, Okur and Karacaer^26^ conducted a study in a military hospital with 254 apparently healthy young individuals (median age: 27 years) and found a prevalence of 10.6%. The study population consisted of male and young individuals, with normal weight, which is likely responsible for the low-NAFLD prevalence. A new retrospective study was conducted by Değertekin et al.^27^ The 10-year data (2007-2016) of 113 239 apparently healthy subjects visiting the checkup clinics were retrospectively analyzed. The result showed a prevalence of 48.3% in Turkey and the NAFLD prevalence was detected as 63.5% in overweight individuals. The highest prevalence in Turkey was in Central Anatolia (57.1%) and in East Anatolia (55.7%). Cappadocia, located in Central Anatolia, has an interesting similarity with our results. Additionally, the study showed a significant increase from 43.5% to 53.1% in NAFLD prevalence in the period between 2007 and 2016.

Our study is the most recent, prospective, population-based study with the largest participation, which provides information on the prevalence of NAFLD in Turkey. Reasons for the high rate (60.1%) of NAFLD in the Cappadocia cohort were that 36.4% of the individuals are overweight, 41.7% are obese, the frequency of concomitant DM, HT, CAD, and HL is high, physical activity is very low both in daily and work life and is related to a sedentary life.

Various studies have revealed a close association between sedentary behavior and metabolic diseases. Prolonged sitting time and decreased physical activity level were positively associated with the prevalence of NAFLD in a large sample of middle-aged Koreans, supporting the importance of reducing time spent sitting in addition to promoting physical activity.^28^ In a Chinese study, they found a higher proportion of NAFLD across the tertiles of sitting time. Multivariate linear regression analyses showed sitting time independently correlated with homeostasis model assessment for insulin resistance (HOMA-IR), alanine aminotransferase, γ-glutamyl transpeptidase, BMI, triglyceride, and the high-sensitive C reactive protein was associated with a higher prevalence of NAFLD (odd ratio [OR] 1.09; 95% CI: 1.04-1.67) after adjusting for confounders.^29^

In our study, NAFLD was also found in 11.4% of individuals with normal weight. In addition, 3.5% of those with HS had a BMI <25. The normal weight HS rate in the entire cohort was 2.1%. In this group, which was named as lean NAFLD, male predominance was present, and the mean age was lower. In the lean NAFLD group, the ultrasonographic HS stage tended to be milder and had less liver size and splenic vein diameter. Lean NAFLD is detected in 10%-20% of Americans and Caucasians.^30^ Akyuz et al^31^ reported this rate as 7.6% in Turkey. In normal weight (lean) NAFL cases, the prevalence of NASH was similar to overweight NAFLD, histological activity score was lower, steatosis and ballooning of hepatocytes were detected less, but the risk of advanced fibrosis was not different. Metabolic syndrome may be an indicator of advanced disease.^32^ In these, 17% advanced liver damage (NASH and >F2 fibrosis) can be observed.^33^ At this point, the clinical importance of screening with TAU and detection of HS again emerges.

Hepatic steatosis was more common in men than women and increased in relation to aging and BMI increase (Figure 2). Liver size, PV and splenic vein diameter increased in direct proportion to HS (Figure 3). A recent study from Turkey by Özmen et al^34^ reported the mean liver length as 149 ± 1.6 mm in normal population. In our study, liver size was found to be 150 ± 9.2 mm. Ultrasonography plays an important role in the evaluation of the PV diameter and flow characteristics in the vessel. Previous studies from different countries reported the mean PV diameter among healthy controls documented as 0.96 ± 0.14 cm and 0.115 ± 0.15 cm also found no significant differences of the mean PV diameter between the males and the females, and age.^35,36^ In our study, the PV diameter was found to be 11 ± 1.1 mm. Although it is difficult to comment on the clinical significance of the increased diameter of the PV and splenic vein, it may be associated with increased intrahepatic resistance caused by adiposity and fibrosis and may be a valuable finding for portal hypertension.

The frequency of DM, HT, HL and the number of births in women were significantly higher in those with HS, and the frequency of diseases other than HL and CAD increased significantly with the stage of HS (Figures 4 and 5). In the regression analysis, it was shown that male gender (HR: 3.2), the presence of HT (HR: 1.5), and high BMI (BMI: 25-30 HR: 9.3, BMI > 30 HR: 75.2) were effective (Table 1).

Studies on obesity in Turkey show that overweight and obesity are common and their frequency increases over time. The largest population-based studies on this subject in Turkey are the Turkish Dia­betes Epidemiology Study (TURDEP)-I and the TURDEP-II studies, which were conducted in 2001 and 2012.^37,38^ The latest TURDEP-II study showed that 32% of the general population in Turkey were obese. TURDEP studies have shown that the prevalence of obesity has increased by 44% in 12 years. In the European Cardiovascular Disease Statistics (ATLAS) study announced in 2017, the rates reported for obesity in Turkish women and men were found to be 35.8% and 22.9%, respectively.^39^ In Western Europe, it is approximately 20%.^3^ As these results show, Turkey is one of the leading countries in Europe in terms of obesity. Similarly, nearly 70% of the US adult population is either obese or overweight.^40^

The prevalence of DM in the Cappadocia cohort was 14%. In a meta-analysis evaluating the prevalence of DM in our country, the prevalence of DM was 13.5% (95% CI: 11.6-15.5%) in the whole group, 14.2% (95% CI: 12.3-16.2%) in the women, and 12.6% (95% CI: 10.5-14.9%) in the men has been reported.^41^ The results of this meta-analysis were similar to ours. Unfortunately, the prevalence of DM is increasing rapidly all over the world and in our country.^37,38^ The most important reasons for the increasing incidence of DM are the increase in overweight and obesity, sedentary lifestyle, and aging of the society.

In a systematic review and meta-analysis evaluating the epidemiological studies on the frequency of HL in the last 15 years in Turkey, the prevalence of hypercholesterolemia was found to be 29.1% (95% CI: 23.6-35.0%) in the general population, 30.2% in women (95% CI: 24.7-36.1%), and 27.8% (95% CI: 22.3-33.6%) in the men.^42^ Since the presence of hypercholesterolemia (8.3%) in the Cappadocia cohort was evaluated based on the reports of the participants, this result may not reflect the reality correctly.

The prevalence of HT in our country was found to be 33.7% in the Türk Erişkinlerindeki Kalp Hastalığı ve Risk Faktörleri (TEKHARF) study and 31.8% in the Turkish Hypertension Prevalence Study.^43,44^ Most recently, the prevalence of HT was found to be 24% in individuals over the age of 15.^45^ The frequency of HT in the Cappadocia cohort was 38.9%. These 3 diseases are the most important metabolic risk factors and are associated with NAFLD.

Our ultrasonography study detected the rate of mass in the liver as 2.5% and showed that 85% of it was hemangioma. The rate of liver cirrhosis was 0.2%. Although HS and MS were so high, the rate of cirrhosis was very low and no associated liver cancer was detected. We think that further studies are needed on this subject.

Gallbladder wall thickness is a parameter that can be accurately determined by TAU, and no pathological thickening of the gallbladder wall was detected in our study.

The incidence of gallstones in the Cappadocia cohort was 7.6%, and it was twice as high in the women as in the men. The incidence of gallstones increased linearly with age and BMI (Figures 6 and 7). After the fifth decade and with excess weight, it increased very clearly. Gallstones were present in approximately one-third of the population over the age of 80 and two-thirds of the obese. The rates of DM, HT, HL, and CAD were significantly higher in those with gallstones. In the regression analysis, female gender (HR: 1.4), increased BMI (BMI: 25-30 HR: 2.1, BMI > 30 HR: 2.9), aging (30-39 age range HR: 1.5, >70 years old HR: 5.8), and HT (HR: 1.4) were found to be the most effective factors in gallstone formation (Table 2).

Among the risks involved in the formation of gallstones, the most well-known are obesity, hyperinsulinemia, and associated DM.^46-48^ Indeed, while 10%-12% of the adult population has gallstones on average, these rates can increase up to 20%-30% in diabetic individuals.^49-52^

Except for 2 studies conducted in 1992 and 1993 to determine the frequency of gallstones in our country, we could not find any comprehensive study. Özütemiz et al^53^ found the prevalence of silent gallstones to be 7.79% in the Aegean region. Beyler et al^54^ in a screening study involving 2188 people from different parts of Turkey, they found an average of 5.25% in the population, 7% in the women and 3.5% in the men. They found that the frequency of gallstones increased with age, and with the number of births and weight in women. A similar situation can be observed in the European continent, with different figures ranging from 5.9% to 21.9% for the frequency of gallstones in large population-based studies.^55-57^ An interesting thing is that although 30 years have passed, the frequency of gallstones in Turkey is the same. Despite the increase in obesity and DM, an increase in the frequency of gallstones was not observed. If we assume that 250 people with cholecystectomy were operated for gallstones, the rate of gallstones in the cohort would be 15.8%. However, it is not possible to conduct a judgment about this topic as we have not investigated the cause for cholecystectomy.

This is because, it is possible that gallstone formation is multifactorial and while not all obese people develop gallstones, not everyone with gallstones is obese. The concentration of cholesterol in the bile, deterioration of gallbladder motility, and increase in bile mucin density are some of the factors in the formation of gallstones. It was determined that gallstones did not form in those who were obese and had normal bile cholesterol saturation. Hepatic acyl-coenzyme A:cholesterol acyltransferase (ACAT) activity may be normal in obese individuals, and it can prevent excessive cholesterol secretion into the bile and thus prevent gallstone formation. In addition, some observational studies have shown that gallstones can occur independently of BMI and insulin resistance.^58^

Gallbladder polyps are mass lesions extending from the gallbladder mucosa to the lumen. Ultrasonography is the most commonly used imaging method in the detection of gallbladder polyps, as in other gallbladder pathologies, and its sensitivity is around 90%. In previous TAU studies, gallbladder polyps were found in 3%-7% of people and 2%-12% in cholecystectomy specimens.^59-62^ In the Cappadocia cohort, the frequency of gallbladder polyps was 2.1%. No difference was found between those with and without polyps in the gallbladder.

Only 1 patient had an image compatible with Caroli’s disease and 1 had a stone in the intrahepatic biliary tract.

Approximately 8.9% of the Cappadocia cohort had cholecystectomy operation. In this study, it is not possible to comment on cholecystectomy, since we do not have an objective information on the indications for cholecystectomy and the results of postsurgical specimen evaluation. The female predominance in those undergoing cholecystectomy was clear.

In our study, no individual with significant pathology regarding pancreatic size and parenchyma structure was found.^63^ Likewise, the spleen and vascular structures were often normal.

One of the strengths of our study is that TAU was applied prospectively in the general population instead of patients seeking health care, and the findings were evaluated. In Turkey, there is no previous study on TAU and GIS-related disease burden in a cohort that could represent the country at this size. The greatest shortcoming of our study manifested itself in the assessment of pancreatic echogenicity. For years, it was thought that pancreatic echogenicity could be variable and that this was normal. We did not pay special attention to pancreatic echogenicity when we planned this study. However, this view has changed drastically recently, and the increase in pancreatic echogenicity has become very important today, as it is accepted as “pancreatic steatosis” and this is seriously associated with metabolic syndrome and cardiovascular risk factors.^64^ It is necessary to eliminate this deficiency in our future studies by evaluating the pancreatic echo in detail.

As a result, in the Cappadocia cohort representing Turkey, the frequency of HS was found to be quite high at a rate of 60.1% in the screening performed with TAU. The incidence of gallstones is 7.6%, and there has been no increase in its frequency within 30 years. It has been determined that both conditions are directly related to gender, HT, BMI increase, and obesity. The results of the Cappadocia cohort located in central Anatolia, where overweight and lack of physical activity are characteristic, and the frequency of concomitant DM, HT, CAD, and HL are high, showed that Turkey is one of the leading countries in the world for NAFLD.

## Figures and Tables

**Table 1.** Regression Analysis of Factors Affecting Hepatic Steatosis

	*P*	HR	95% CI for HR	
			Lower	Upper
Gender (male)	<.001	3.209	2.580	3.991
HT	<.001	1.530	1.232	1.900
BMI (<25)	<.001			
BMI (25-29.9)	<.001	9.382	6.874	12.803
BMI (>30)	<.001	72.244	51.142	102.053

BMI, body mass index; HT, hypertension.

**Table 2.** Regression Analysis of Factors Affecting the Presence of Gallstones

	*P*	HR	95% CI for HR	
			Lower	Upper
Gender (female)	.036	1.432	1.024	2.002
BMI (<25)	.002			
BMI (25-29.9)	.014	2.192	1.172	4.097
BMI (>30)	.001	2.946	1.592	5.451
HT	.037	1.422	1.022	1.980
Age group (under 30)—Reference	<.001			
Age group (30-39)	.416	1.592	0.519	4.878
Age group (40-49)	.208	2.009	0.678	5.950
Age group (50-59)	.065	2.706	0.939	7.799
Age group (60-69)	.036	3.160	1.076	9.276
Age group (>70)	.002	5.896	1.971	17.637

BMI, body mass index; HT, hypertension.

## References

[1] Sezgin O Akpınar H Özer B Törüner M Bal K Bor S . Population-based assessment of gastrointestinal symptoms and diseases: Cappadocia Cohort, Turkey. Turk J Gastroenterol. 2019;30(12):1009 1020. (10.5152/tjg.2019.19882) 31854305 PMC6924608

[2] Ural D Kılıçkap M Göksülük H , et al. Data on prevalence of obesity and waist circumference in Turkey: systematic review, meta-analysis and meta-regression of epidemiological studies on cardiovascular risk factors. Turk Kardiyol Dern Ars. 2018;46(7):577 590. (10.5543/tkda.2018.62200) 30391987

[3] Berghöfer A Pischon T Reinhold T Apovian CM Sharma AM Willich SN . Obesity prevalence from a European perspective: a systematic review. BMC Public Health. 2008;8:200. (10.1186/1471-2458-8-200) PMC2441615 18533989

[4] Yu XZ Yang YN Li JG . Application of ultrasound in the diagnosis of gastrointestinal tumors. Eur J Inflamm. 2020;18:1 8. (10.1177/2058739220961194)

[5] Onodera H, Chida N, Abe M , et al. Ultrasonic mass survey for liver, biliary tract and pancreatic diseases: a detection of gallbladder diseases. J Gastroent Mass Survey. 1987;74:41 47.

[6] Ünal S Aşcıoğlu S Demirkazık A , et al. Baseline data of a prospective cohort study. Cappadocia cohort study, Turkey. Turk J Public Health. 2018;16(3):190 203.

[7] Johansson G Westerterp KR . Assessment of the physical activity level with two questions: validation with doubly labeled water. Int J Obes (Lond). 2008;32(6):1031 1033. (10.1038/ijo.2008.42) 18392036

[8] Niederau C Sonnenberg A Müller JE Erckenbrecht JF Scholten T Fritsch WP . Sonographic measurements of the normal liver, spleen, pancreas, and portal vein. Radiology. 1983;149(2):537 540. (10.1148/radiology.149.2.6622701) 6622701

[9] Charatcharoenwitthaya P Lindor KD . Role of radiologic modalities in the management of non-alcoholic steatohepatitis. Clin Liver Dis. 2007;11(1):37 54. (10.1016/j.cld.2007.02.014) 17544971

[10] Mazhar SM Shiehmorteza M Sirlin CB . Noninvasive assessment of hepatic steatosis. Clin Gastroenterol Hepatol. 2009;7(2):135 140. (10.1016/j.cgh.2008.11.023) 19118644 PMC6658187

[11] Lupşor-Platon M Stefănescu H Mureșan D , et al. Noninvasive assessment of liver steatosis using ultrasound methods. Med Ultrason. 2014;16(3):236 245. (10.11152/mu.2013.2066.163.1mlp) 25110765

[12] Finberg HJ Birnholz JC . Ultrasound evaluation of the gallbladder wall. Radiology. 1979;133(3 Pt 1):693 698. (10.1148/133.3.693) 504650

[13] Dewbury KC . Visualisation of normal biliary ducts with ultrasound. Br J Radiol. 1980;53(632):774 780. (10.1259/0007-1285-53-632-774) 7437688

[14] Emamian SA Nielsen MB Pedersen JF Ytte L . Kidney dimensions at sonography: correlation with age, sex, and habitus in 665 adult volunteers. AJR. 1993;160(1):83 86. (10.2214/ajr.160.1.8416654) 8416654

[15] Creagh-Barry M Adam EJ Joseph AEA . The value of oblique scan in the ultrasonic examination of the abdominal aorta. Clin Radiol. 1986;37(3):239 241. (10.1016/s0009-9260(86)80325-7) 3519051

[16] Saverymuttu SH Joseph AE Maxwell JD . Ultrasound scanning in the detection of hepatic fibrosis and steatosis. Br Med J (Clin Res Ed). 1986;292(6512):13 15. (10.1136/bmj.292.6512.13) PMC1338970 3080046

[17] Hamaguchi M Kojima T Itoh Y , et al. The severity of ultrasonographic findings in nonalcoholic fatty liver disease reflects the metabolic syndrome and visceral fat accumulation. Am J Gastroenterol. 2007;102(12):2708 2715. (10.1111/j.1572-0241.2007.01526.x) 17894848

[18] Mustapic S Ziga S Matic V , et al. Ultrasound grade of liver steatosis is independently associated with the risk of metabolic syndrome. Can J Gastroenterol Hepatol. 2018;2018:8490242. (10.1155/2018/8490242) PMC6126110 30211140

[19] Li Q Dhyani M Grajo JR Sirlin C Samir AE . Current status of imaging in nonalcoholic fatty liver disease. World J Hepatol. 2018;10(8):530 542. (10.4254/wjh.v10.i8.530) 30190781 PMC6120999

[20] Younossi Z Tacke F Arrese M , et al. Global perspectives on non-alcoholic fatty liver disease and non-alcoholic steatohepatitis. Hepatology. 2019;69(6):2672 2682. (10.1002/hep.30251) 30179269

[21] Estes C Anstee QM Arias-Loste MT , et al. Modeling NAFLD disease burden in China, France, Germany, Italy, Japan, Spain, United Kingdom, and United States for the period 2016-2030. J Hepatol. 2018;69(4):896 904. (10.1016/j.jhep.2018.05.036) 29886156

[22] Perumpail BJ Khan MA Yoo ER Cholankeril G Kim D Ahmed A . Clinical epidemiology and disease burden of nonalcoholic fatty liver disease. World J Gastroenterol. 2017;23(47):8263 8276. (10.3748/wjg.v23.i47.8263) 29307986 PMC5743497

[23] Eguchi Y Hyogo H Ono M , et al. Prevalence and associated metabolic factors of nonalcoholic fatty liver disease in the general population from 2009 to 2010 in Japan: a multicenter large retrospective study. J Gastroenterol. 2012;47(5):586 595. (10.1007/s00535-012-0533-z) 22328022

[24] Younossi ZM Koenig AB Abdelatif D Fazel Y Henry L Wymer M . Global epidemiology of nonalcoholic fatty liver disease-Meta-analytic assessment of prevalence, incidence, and outcomes. Hepatology. 2016;64(1):73 84. (10.1002/hep.28431) 26707365

[25] Celebi S Ataseven H Mengucuk E Deveci SH Acık Y Bahcecioglu İH . Epidemic features of nonalcoholic fatty liver in urban community of Elazıg. Akad Gastroenteroloji Derg. 2006;5:41 46.

[26] Okur G Karacaer Z . The prevalence of non-alcoholic fatty liver disease in healthy young persons. North Clin Istanb. 2016;3(2):111 117. (10.14744/nci.2016.28199) 28058397 PMC5206460

[27] Değertekin B Tozun N Demir F , et al. The changing prevalence of non-alcoholic fatty liver disease (NAFLD) in Turkey in the last decade. Turk J Gastroenterol. 2021;32(3):302 312. (10.5152/tjg.2021.20062) 34160360 PMC8975521

[28] Ryu S Chang Y Jung HS , et al. Relationship of sitting time and physical activity with non-alcoholic fatty liver disease. J Hepatol. 2015;63(5):1229 1237. (10.1016/j.jhep.2015.07.010) 26385766

[29] Wei H Qu H Wang H Deng H . Associations between sitting time and non-alcoholic fatty liver diseases in Chinese male workers: a cross-sectional study. BMJ Open. 2016;6(9):e011939. (10.1136/bmjopen-2016-011939) PMC5020753 27609847

[30] Younes R Bugianesi E . NASH in lean individuals. Semin Liver Dis. 2019;39(1):86 95. (10.1055/s-0038-1677517) 30654392

[31] Akyuz U Yesil A Yilmaz Y . Characterization of lean patients with nonalcoholic fatty liver disease: potential role of high hemoglobin levels. Scand J Gastroenterol. 2015;50(3):341 346. (10.3109/00365521.2014.983160) 25540973

[32] Leung JC Loong TC Wei JL , et al. Histological severity and clinical outcomes of nonalcoholic fatty liver disease in nonobese patients. Hepatology. 2017;65(1):54 64. (10.1002/hep.28697) 27339817

[33] Fracanzani AL Petta S Lombardi R , et al. Liver and cardiovascular damage in patients with lean nonalcoholic fatty liver disease, and association with visceral obesity. Clin Gastroenterol Hepatol. 2017;15(10):1604 1611.e1. (10.1016/j.cgh.2017.04.045) 28554682

[34] Özmen Z Aktaş F Özmen ZC Almus E Demir O . Ultrasound measurement of liver longitudinal length in a North Anatolian population: a community-based study. Niger J Clin Pract. 2018;21(5):653 657. (10.4103/njcp.njcp_68_17) 29735868

[35] Luntsi G Sani M Zira JD Ivor NC Garba SH . Sonographic assessment of the portal vein diameter in apparently healthy adults in a Northern Nigerian population. Afr Health Sci. 2016;16(4):1163 1168. (10.4314/ahs.v16i4.35) 28479910 PMC5398464

[36] Hawaz Y Admassie D Kebede T . Ultrasound assessment of normal portal vein diameter in Ethiopians done at Tikur Anbessa Specialized Hospital. East Cent Afr J Surg. 2012;17:90 93.

[37] Satman I Yilmaz T Sengül A , et al. Population-based study of diabetes and risk characteristics in Turkey: results of the Turkish diabetes epidemiology study (TURDEP). Diabetes Care. 2002;25(9):1551 1556. (10.2337/diacare.25.9.1551) 12196426

[38] Satman I Omer B Tutuncu Y , et al. Twelve-year trends in the prevalence and risk factors of diabetes and prediabetes in Turkish adults. Eur J Epidemiol. 2013;28(2):169 180. (10.1007/s10654-013-9771-5) 23407904 PMC3604592

[39] Timmis A Townsend N Gale C , et al. European Society of Cardiology: cardiovascular disease statistics 2017. Eur Heart J. 2018;39(7):508 579. (10.1093/eurheartj/ehx628) 29190377

[40] Ogden CL Carroll MD Fryar CD Flegal KM . Prevalence of obesity among adults and youth: United States, 2011-2014. NCHS Data Brief. 2015;(219):1 8. 26633046

[41] Yılmaz MB Kılıçkap M Abacı A , et al. Temporal changes in the epidemiology of diabetes mellitus in Turkey: A systematic review and meta-analysis. Turk Kardiyol Dern Ars. 2018;46(7):546 555. (10.5543/tkda.2018.88225) 30391984

[42] Kayıkcıoglu M Tokgözoğlu L Kılıçkap M , et al. Data on prevalence of dyslipidemia and lipid values in Turkey: systematic review and meta-analysis of epidemiological studies on cardiovascular risk factors. Turk Kardiyol Dern Ars. 2018;46(7):556 574. (10.5543/tkda.2018.23450) 30391985

[43] Onat A Şenocak M Örnek E , ve ark. Türkiye’de erişkinlerde kalp hastalığı ve risk faktörleri sıklığı taraması: 5. Hipertansiyon ve sigara içimi. Türk Kardiyol Dern Arş. 1991;19:169 177.

[44] Altun B Arıcı M Nergizoğlu G , et al. Prevalence, awareness, treatment and control of hypertension in Turkey (the PatenT study) in 2003. J Hypertens. 2005;23(10):1817 1823. (10.1097/01.hjh.0000176789.89505.59) 16148604

[45] Pamukcu B . Profile of hypertension in Turkey: from prevalence to patient awareness and compliance with therapy, and a focus on reasons of increase in hypertension among youths. J Hum Hypertens. 2022;36(5):437 444. (10.1038/s41371-020-00480-6) 33462387

[46] Scragg RKT Calvert GD Oliver JR . Plasma Lipids and insulin in gall stone disease: a case control study. Br Med J (Clin Res Ed). 1984;289(6444):521 525. (10.1136/bmj.289.6444.521) PMC1442729 6432171

[47] Scragg RKT McMichael AJ Baghurst PA . Diet, alcohol and relative weight in gall stone disease; a case control study. Br Med J (Clin Res Ed). 1984;288(14):1113 1119. 10.1136/bmj.288.6424.1113 PMC1441375 6424754

[48] Jørgensen T . Gallstones in a Danish population. Relation to weight, physical activity, smoking, coffee consumption and diabetes mellitus. Gut. 1989;30(4):528 534. (10.1136/gut.30.4.528) 2785475 PMC1434027

[49] Bennion CJ . Risk factors for the development of cholelithiasis in man. NEjm. 1978;229(22):1221 1227. 10.1056/NEJM197811302992205 362198

[50] Pagliarulo M Fornari F Fraquelli M , et al. Gallstone disease and related risk factors in a large cohort of diabetic patients. Dig Liver Dis. 2004;36(2):130 134. (10.1016/j.dld.2003.10.007) 15002821

[51] Diehl AK . Cholelithiasis and the insulin resistance syndrome. Hepatology. 2000;31(2):528 530. (10.1002/hep.510310238) 10655281

[52] Ruhl CE Everhart JE . Association of diabetes, serum insulin, and C-peptide with gallbladder disease. Hepatology. 2000;31(2):299 303. (10.1002/hep.510310206) 10655249

[53] Özütemiz Ö Batur Y Özgüven Ö . Ege bölgesinde sessizsafra taşı prevalansı. Klin Gelişim. 1992;5:1737 1741.

[54] Beyler AR Uzunalimoğlu Ö Gören A Özden A , et al. Türkiyede normal Populasyonda Safra kesesi taşı Prevelansı. Turk J Gastroenterol. 1993;4(3):434 437.)

[55] Aerts R Penninckx F . The burden of gallstone disease in Europe. Aliment Pharmacol Ther. 2003;18(suppl 3):49 53. (10.1046/j.0953-0673.2003.01721.x) 14531741

[56] American Gastroenterological Association. The Burden of Gastrointestinal Diseases. Bethesda, (MD):The American Gastroenterological Association; 2001.

[57] Sampliner RE Bennett PH Comess LJ Rose FA Burch TA . Gallbladder disease in Pima Indians: demonstration of high prevalence and early onset by cholecystography. N Engl J Med. 1970;283(25):1358 1364. (10.1056/NEJM197012172832502) 5481754

[58] Cortés VA Barrera F Nervi F . Pathophysiological connections between gallstone disease, insulin resistance, and obesity. Obes Rev. 2020;21(4):e12983. (10.1111/obr.12983) 31814283

[59] Myers RP Shaffer EA Beck PL . Gallbladder polyps: epidemiology, natural history and management. Can J Gastroenterol. 2002;16(3):187 194. (10.1155/2002/787598) 11930198

[60] Lee KF Wong J Li JC Lai PB . Polypoid lesions of the gallbladder. Am J Surg. 2004;188(2):186 190. (10.1016/j.amjsurg.2003.11.043) 15249249

[61] Terzi C Sökmen S Seçkin S Albayrak L Uğurlu M . Polypoid lesions of the gallbladder: report of 100 cases with special reference to operative indications. Surgery. 2000;127(6):622 627. (10.1067/msy.2000.105870) 10840356

[62] Jørgensen T Jensen KH . Polyps in the gallbladder. A prevalence study. Scand J Gastroenterol. 1990;25(3):281 286. (10.1080/00365521.1990.12067104) 2320947

[63] Khammas ASA Mahmud R . Ultrasonographic measurements of the liver, gallbladder wall thickness, inferior Vena cava, portal vein and pancreas in an urban region, Malaysia. J Med Ultrasound. 2021;29(1):26 31. (10.4103/JMU.JMU_53_20) 34084713 PMC8081096

[64] Sezgin O Yaraş S Özdoğan O . Pancreatic steatosis is associated with both metabolic syndrome and pancreatic stiffness detected by ultrasound elastography. Dig Dis Sci. 2022;67(1):293 304. (10.1007/s10620-021-06844-3) 33651254

